# “Back to Where They Were”: The Socio-Discursive Representation of Transgender Sex Workers and Urban Space in a Television News Report

**DOI:** 10.3389/fsoc.2021.633699

**Published:** 2021-04-12

**Authors:** Matías Soich

**Affiliations:** ^1^ National Scientific and Technical Research Council, Buenos Aires, Argentina; ^2^ Literature Department, University of Buenos Aires, Buenos Aires, Argentina; ^3^ Mocha Celis Civil Association, Buenos Aires City, Argentina

**Keywords:** critical discourse analysis, socio-discursive representations, social exclusion, city of Buenos Aires, urban space, prostitution, transgender, transvestites

## Abstract

Despite significant advances in recent years, Argentina’s transgender community still faces structural social exclusion. For a vast majority of transvestites and transgender women, early expulsion from the family home and the educational system results in having to resort to prostitution as their only option for surviving. Police edicts and other similar devices are used to penalize prostitution and persecute transgender people in public places, showing that prejudice and violence against their identities also manifest in the control of urban space. Here I present the results of an in-depth qualitative linguistic analysis of a 2018 television news report about the temporary relocation of the transgender sex workers from their usual location in the *Bosques de Palermo*, the biggest public park in the City of Buenos Aires. The theoretical frame is Critical Discourse Analysis and the methodology is inductive and qualitative. The analysis centers on the linguistic resources that define the socio-discursive representation about the transgender sex workers in relation with urban space and the city’s government. The bases of the analysis are the Synchronic-Diachronic Method for the Linguistic Analysis of Texts and the Method of Converging Linguistic Approaches. These methods revealed, in the first place, that the transvestites and transgender women are represented as mere occupants of public space through their close association with the discursive category of *Space*. In the second place, they are represented as fundamentally passive in relation to the Government of the City of Buenos Aires; while, at the same time, the government’s responsibility for their displacement is systematically mitigated. Finally, the lack of work alternatives to prostitution for the transgender community is naturalized through the persistent association of the discursive categories connected with transgender people, prostitution and urban space. If we compare these results with those of previous research, we can see that these discursive features—none of which challenge the status quo—remain one of the basic components of the socio-discursive representation of transgender people elaborated by the mainstream media.

## Introduction

This work deals with the socio-discursive representation elaborated in a 2018 television news report about the transgender and transvestite sex workers^1^ in the City of Buenos Aires. This report was chosen for analysis for two main reasons: the social and political influence wielded by the channel that produced and aired it, and the social relevance of its topic. The theoretical frame is Critical Discourse Analysis, which means that the focus is on the systematical study of the text’s linguistic components, which are interpreted in the light of their social and political context, with the ultimate goal of creating knowledge that contributes to the solution of social problems connected with discrimination, inequality and power abuse (van Dijk, 1999; Pardo, 2008). The specific topic of the news report is the temporary relocation of the transgender sex workers within Buenos Aires’ biggest public park. It thus targets public space as a site of urban conflict (Boy, 2015), with gender identity becoming the main variable of governmental control over one of Argentina’s most excluded and discriminated social groups.

The paper is organized as follows. In *The Transgender Community in the City of Buenos Aires*, I provide a succinct description of the situation of the transgender community in the City of Buenos Aires, highlighting those aspects directly related to the news report’s topic. In *Theoretical and Methodological Frame*, I describe the methodological frame and explain the analytical methods that were applied to the corpus. In *Corpus*, I describe the corpus in terms of the search and selection process, and of the components of the communicative situation. In *Linguistic Analysis* and its subsections, I present the main results of the linguistic analysis of the corpus, providing examples and explanations for each particular aspect of the socio-discursive representation at stake. Finally, in *Conclusion*, I summarize the findings and offer a brief final reflection.

## The Transgender Community in the City of Buenos Aires

Worldwide, the human rights scenario is far worse for transgender people than for the cisgender (i.e., not transgender) majority. In Argentina, studies conducted by different transgender organizations (Berkins and Fernández, 2005; Berkins, 2007; Fundación Huésped and ATTTA, 2014; Akahatá et al., 2016) have exposed the violence and dire life conditions faced by this social group due to the systematical exclusion and discrimination promoted by the State and society. Despite offering more favorable opportunities (especially for work, health access and exposure to discrimination), the City of Buenos Aires, the country’s capital and richest district, shows a worrying violation of transgender human rights. A study conducted in 2016 with 169 transvestites and transgender women and 33 transgender men (Ministerio Público de la Defensa and Bachillerato Popular Travesti-Trans Mocha Celis, 2017) revealed that 59% of the transvestites and transgender women in the city had not completed the minimum mandatory educational level (high school), and only 9% had access to the formal labor market. 65% of them lived in rented room in hotels or abandoned houses, and 74.6% had experienced some form of violence (insults, robberies, physical aggression and sexual abuse, among others), especially in the streets, police stations and schools.

For the transgender community, and especially for transvestites and transgender women, prostitution is the only option for generating income after their early expulsion from the family home. The same study showed that prostitution was the main occupation of 70% of the transvestites and transgender women, 87% of whom declared that they would abandon that activity if offered a formal job. Age and educational level are directly correlated with this: the higher those two variables, the lower the percentage of transgender women who turn to prostitution for a living.^2^

In the City of Buenos Aires, the social struggle over the presence of transgender sex workers (and of transgender women in general) in public spaces triggered the emergence of the first transvestite and transgender activist organizations during the decade of 1990 (Berkins, 2003; Fernández, 2004). These organizations fought against the penalization of prostitution and of transgender identities. Since 1949, two sections of the city’s edicts (2° F and 2° H) had allowed the police to arbitrarily sanction and detain anyone who “exhibit themselves in the public space wearing clothes of the opposite sex” and “people of either sex that publicly incite or offer themselves for the carnal act”. These edicts enabled criminalization of people with different gender identities and sexual orientations, such as transvestites and homosexuals, regardless of their relation with prostitution. When the city became autonomous in 1998, the 2° F and 2° H sections were abolished, and the city’s new legislative body sanctioned the more liberal Code of Urban Cohabitation, which did not penalize transvestism and prostitution in public spaces. However, due to the pressure of neighbor associations and the Catholic Church, article 71, titled “Alteration of public tranquility,” was added later that same year. This article did not penalize transvestism per se, but condemned prostitution, exhibition of underwear and nudity in public spaces. The next year, a presidential decree re-established the old edicts and article 71 was modified to explicitly ban prostitution. Finally, in 2004, the Code was modified again, with article 81 now penalizing “the offer and demand of sex in public spaces” (Berkins and Fernández, 2005, pp. 39–66).

These comings and goings in legal reform reflect a complex urban conflict that involved the transvestite and transgender sex workers, the city’s neighbor associations, government officials, the police and the Catholic Church, all of which “emitted discourses about the legitimate and illegitimate uses of public space, about who deserved to live in the city, and about the transvestites and their bodies” (Boy, 2015, p. 177). At that time, the circulation of these discourses in the mass media marked a turning point in the visibilization of transgender people, although the treatment they received in the media was anything but respectful, with trivialization of their identities and verbal aggression being the deplorable norm (Berkins, 2003; Naty Menstrual, 2009; Wayar, 2009; Vásquez Haro, 2012).

The urban conflict around transgender people and public space was particularly noticeable in Palermo, one of the neighborhoods with more transgender sex workers.^3^ With moral arguments that condemned prostitution and transvestism as an “obscenity,” the cisgender neighbors of Palermo demanded first the banning of prostitution and then the creation of a special zone—called “the red zone”—where transvestites and transgender women would offer their sexual services away from homes, schools and churches. After many conflicts and negotiations that were abundantly covered by the media, in 2005 the transvestites and transgender sex workers moved to the spot of the *Bosques de Palermo* (Palermo Woods, the city’s biggest public park) known as *El Rosedal* (the Rose Garden). The new location, however, did not please the cisgender neighbors and, a few years later, the sex workers had to move again to another part of the *Bosques* called Florencio Sánchez Square, where they still work today. During this process, the Government of the City of Buenos Aires promised to the transgender organizations several measurements to enhance their work conditions, such as installing chemical bathrooms, building speed bumps and improving public lighting. These promises were only partly fulfilled.

In August 2018, due to the upcoming realization of the Summer Youth Olympic Games near Florencio Sánchez Square, the Government of the City temporarily relocated the transgender sex workers to another part of the *Bosques* known as the Planetarium. This was agreed after negotiations with different transgender and social organizations, including the Argentinean LGBT Federation (FALGBT), the Argentinean Transvestites, Transsexuals and Transgender Association (ATTTA), the Trans House, the Gondolín Hotel Civil Association, and the Argentinean Association of Women Prostitutes (AMMAR) (Clarín, 2018). Unlike the negotiations that began in the late 1990s and led to the transgender sex workers moving to *El Rosedal* in 2005, this episode did not involve public demonstrations or conflicts. Nevertheless, it showed that, even six years after the passing of the National Gender Identity Law in 2012, governmental decisions about the use of urban space still considered transgender bodies an “obscenity” that must be removed from the public—and international—eye.^4^

## Theoretical and Methodological Frame

The theoretical frame of this work is Critical Discourse Analysis (Fairclough, 1992; van Dijk, 2001a), particularly as expressed in the Latin American Network for the Analysis of the Discourse of Extreme Poverty (REDLAD) (Resende and Ramalho, 2006; García Da Silva, 2007; Pardo Abril, 2007; Pardo, 2008; Montecino Soto, 2010). This means that the primary focus is on studying how the ways in which language is used take part in social problems like inequity, power abuse and discrimination. In this regard, Critical Discourse Analysis is not defined as an exclusive and closed method for studying those problems, but as a critical perspective to be adopted regardless of the methods chosen for the linguistic analysis of texts (van Dijk, 2001b). It is important, however, that the methods provide solid linguistic evidence, so that researchers can make their claims on the basis of observable linguistic and discursive data, rather than make commentaries loosely based on the texts’ content (Pardo et al., 2018, p. 94).

Discourse as analytical material is a complex phenomenon that cannot be reduced to formal linguistic structures. It requires a holistic interpretation that also takes into account contextual and social aspects of the communicative situation. For this reason, this research is positioned in the interpretativist paradigm (Guba and Lincoln, 1994) and the methodology is fundamentally qualitative with data triangulation (Pardo, 2011).

Research in Critical Discourse Analysis is essentially interdisciplinary, because studying the role of language in use in social problems requires a deep comprehension of different (social, political, historical, etc.) aspects of the context that exceed the field of Linguistics. Thus, although this particular work concentrates on showing the results of the linguistic analysis, the contextualization and interpretation of the data draws heavily from social studies about gender and sexuality, particularly South American transvestite theory (Berkins, 2003; Berkins and Fernández, 2005; Ministerio Público de la Defensa and Bachillerato Popular Travesti-Trans Mocha Celis, 2017; Wayar, 2018). It is worth mentioning that, in the last decades in Argentina, transgender issues have gradually become a focus of attention for the social sciences, resulting in different works that analyze discursive aspects and representations of transgender identities from a qualitative point of view. However, most of these works are mainly concerned with the texts’ content and semantic features, and do not present a systematized analysis of linguistic forms and strategies such as the one we intend to offer here (Soich, 2017, p. 109).

The main analytical method is the Synchronic-Diachronic Method for the Linguistic Analysis of Texts (henceforth SDMLAT) (Pardo, 1994; Pardo, 2011). This method “accounts both for the categories required by any basic theory (Glaser and Strauss 1967) and for the linguistic properties through which such categories materialize in a given text” (Pardo and Lorenzo-Dus, 2010, p. 258). The SDMLAT involves two basic category types that show how social subjects arrange and give meaning to the world through discourse: the grammaticalized and the semantic-discursive categories. Grammaticalized categories have a high frequency of use in different discursive genres and therefore are mandatory. Semantic-discursive categories are text-specific: they depend on the particular set of meanings that are constructed in each discourse and, therefore, are not mandatory. While grammaticalized categories constitute a finite list, semantic-discursive categories are virtually infinite.

According to Pardo (2011, pp. 67–68), the grammaticalized categories are:

- *Speaker-Protagonist (S-P):* this category is constructed by any pronominal persons or nominal referents that assume the central argument in the text. In other words, it incarnates the text’s “central point of view,” from which other arguments can be developed.
- *Value Nexus 1 (VN1):* this category manifests the actions and states associated with the Speaker-Protagonist. It can be instantiated by finite and non-finite verbs as well as by nominalizations.
- *Actor/s:* this category or categories are constructed by any pronominal persons or nominal referents that assume arguments opposed to the one developed in the Speaker-Protagonist category.
- *Value Nexus 2, 3 … (VN2, VN3 … ):* they manifest the actions and states associated with the Actor/s. They can be instantiated by finite and non-finite verbs as well as by nominalizations.
- *Time* and *Space:* these two categories construct the temporal and spatial orientation inherent to any text.
- *Pragmatic Operator (PO):* this category has several functions, such as involving the hearer or reader, pointing out how an utterance^5^ must be interpreted and connecting utterances or utterance parts. It can be instantiated by different constructions and word types, such as vocatives, interjections and connectors, among others.
- *Negation:* it is a “floating” category, in the sense that it can appear, for example, negating a verb (I did *not* go) or a word (*un*necessary), and it is not mandatory to the same degree than the others.

As was said, semantic-discursive categories are text-specific and therefore, unlike grammaticalized categories, they vary depending on the discursive genre and the context. Some examples of semantic-discursive categories based on previous research are *Gender identity*, *Education* and *Work* in life stories (Soich, 2017), *Insecurity* in news from the written press (Molina, 2015), *Social policies* in legal norms (Marchese, 2017) and *Delinquency* and *Family* in television news chronicles (Pardo, 2014). Semantic-discursive categories are named inductively after particular sets of meanings that appear throughout the text and refer (directly or indirectly) to a specific topic or phenomenon (Pardo, 2011, p. 71).

An important underlying concept of SDMLAT is argumentation as a basic principle of language. This means that regardless of the discursive genre, all texts possess a certain degree of argumentation, which manifests in the presence of strategies or minimal argumentational features that make the text “move forward” until it attains its communicative goal (Lavandera, 1992; Pardo, 2011). The *Speaker-Protagonist* and *Actor* categories of the SDMLAT take this principle to the methodological plane by embodying different chains of reasoning that respond to each other as the text unfolds. In other words, each of these categories represents a distinct argumentative paradigm. These paradigms, in turn, represent the internalized voices that make up the dialogism inherent to any text (Bakhtin, 1981; Pardo, 2011, p. 68).

The application of the SDMLAT takes the concrete form of a table, with each column representing a different category (see Table 1). All lexical elements in the text are classified in either a grammaticalized or a semantic-discursive category, using as many rows as necessary to respect their order of appearance in the utterance. This way, when categorization is concluded, the text can be read by following the table’s rows from left to right and from top to bottom. This enables two complementary readings that give the method its name: a synchronic, “horizontal” reading, which follows the unfolding of each utterance through the different categories; and a diachronic, “vertical” reading, which follows each category as it is progressively developed and charged with meaning throughout the text. Combined, both readings allow a detailed analysis of the linguistic resources through which the discursive categories are instantiated. This, in turn, provides concrete linguistic evidence to study how discursive representations are constructed in the text (Pardo, 2011; Marchese, 2012).

**TABLE 1:** The two readings in the synchronic-diachronic method. 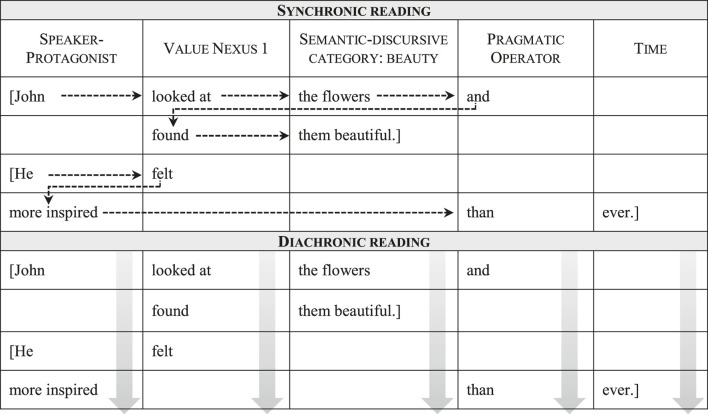

The SDMLAT is continued through the Method of Converging Linguistic Approaches (henceforth MCLA) (Marchese, 2011; Marchese, 2012; Marchese, 2016), which amplifies it by adding analytical phases to study different linguistic aspects that converge in the construction of meaning. In this sense, SDMLAT becomes the first phase of MCLA. In this phase, the analysis concentrates on determining which discursive categories appear in the text and how.

For this paper, besides the MCLA’s first analytical phase (or SDMLAT), I have also adopted its second phase, which studies how information is organized in the utterance.^6^ To do so, the method resorts to the theories of information hierarchization and tonalization (Firbas, 1964; Lavandera, 1986; Pardo, 1994; Pardo, 1996; Pardo, 2011). The theory of information hierarchization studies how the order in which information appears in the utterance relates to that utterance’s communicative goal. From this theory, I will retain the concept of *focus*, defined as the section of the utterance that contains the information necessary to complete that utterance’s communicative goal. In Spanish and other romance languages, this coincides with the final section of the utterance (Pardo, 2011, p. 35). In other words, the information placed at the end of the utterance is the one most important in relation to its communicative goal, and this information is said to be placed in the focus position or *focalized*.

The theory of information tonalization takes the study of hierarchization to a “smaller” plane within the utterance. Its two fundamental concepts are those of *reinforcing* and *mitigating resources*, which are defined as any linguistic resources (for example, the active or passive voice, verb tenses and persons, adverbial constructions, lexical choices, word order, etc.) that respectively strengthen or weaken a certain argument. These two notions are mutually relative and complementary.

All these concepts are gathered in the second phase of the MCLA in order to analyze, in the first place, which discursive categories in the SDMLAT contain the information most relevant for the text’s communicative goal. These are called the *focus categories*. A category becomes a focus category when it is the last category in an utterance. This means that the information that appears in that category is the one that fulfills that utterance’s communicative goal (Marchese, 2011). The observation of the focus categories in a text provides statistic data, such as which focus categories are predominant and in what proportion. As this research is positioned in the interpretativist paradigm, statistic data is always qualitatively interpreted.

In the second place, the second phase of the MCLA allows us to detect, mark and interpret the reinforcing and mitigating resources that appear in different discursive categories. Studying these linguistic resources is key to understanding how the argumentative paradigms that make up the text are constructed with varying degrees of emphasis.

Regarding this second phase, I have also used Halliday and Matthiessen’s (2004) process types classification (material, mental, relational, verbal, behavioral and existential processes) as a means of describing the lexical elements that instantiate the *Nexus Value* categories. The use of specific process types to express actions and states in these categories was interpreted in terms of reinforcing or mitigating resources that affect the representation of the different *Actors* in terms of agency. In this sense, for example, material or verbal processes tend to construct the *Actors* with a higher degree of agency, and this is usually interpreted as a reinforcing resource; while relational or existential processes tend to reduce the degree of agency and, therefore, are usually interpreted as mitigating resources.

The approach inherent to the SDMLAT and the MCLA meets the goals of Critical Discourse Analysis for several reasons. In the first place, it allows working with the products of language in use, i.e., concrete texts, as complete units of analysis, instead of focusing only on fragments, text selections or ideal sentences (as is the case in positivist linguistic studies such as the ones from Labovian Sociolinguistics, or even in other interpretativist studies from Discourse Analysis) (Pardo, 2013). In the second place, context is also incorporated as an intrinsic component of those units of analysis, with the grammaticalized and semantic-discursive categories providing a theoretical and methodological basis for the nexus between language and the social (Pardo, 2011, p. 28). In the third place, these methods allow to inductively obtain concrete linguistic evidence about specific social phenomena while, at the same time, reflecting on the nature of language in use, thus tracing a “virtuous circle” between analysis and theory (Pardo et al., 2018). Finally, both the SDMLAT and the MCLA are methods created in Latin America and were designed with Latin American contexts and problems in mind, in a conscious effort to question and critically reinterpret received traditions from a decolonizing perspective (Pardo et al., 2020).

This said, it is important to mark that, in accordance with the interdisciplinary spirit of Critical Discourse Analysis, the SDMLAT is open to incorporating other theories and methods, even if they come from other areas of linguistic studies. This, of course, does not mean that any theory can be simply and directly incorporated into the SDMLAT, as this requires a degree of critical examination and re-interpretation of the basic theoretical assumptions at stake. An example of the expansion of the SDMLAT is the systematic inclusion of the process types classification from Systemic Functional Grammar (Marchese, 2011); another is the incorporation of the Theory of Conceptual Metaphor (Soich, 2017; Pardo et al., 2018;Pardo et al. 2020). Thus, this work’s analytical approach is not incompatible, but potentially complementary with other theories and methods from the branch of functional linguistics (for example, the theory of speech acts or the theory of appraisal).

## Corpus

The corpus consists of a news report titled “Las chicas trans corridas por los Juegos Olímpicos de Buenos Aires” (“The trans girls displaced by/because of the Buenos Aires Olympic Games”).^7^ It was aired on September 26, 2018 at 9 pm on *Telenoche*, the central news program of *El Trece*, one of the most watched free-to-air television networks in Argentina. Ideologically aligned to the right, *El Trece* belongs to the corporate group Clarín, which throughout its history has had a significant influence on Argentina’s social and political life.^8^ The same day it aired, the report was also uploaded to *El Trece*’s official YouTube channel, where it had considerable impact: to date, it has over two million views, 15,143 likes and 4,599 comments.^9^ The linguistic analysis was carried out on this uploaded video, which lasts 9’44”.

The search that led to this video was made on August 6, 2020 in YouTube, using as keywords “trans,” “city” and “Buenos Aires”. The first ten results are listed in their order of appearance in Table 2. Videos consisting of street interviews about transgender prostitution are marked with an asterisk; as can be seen, they amount to half of these results. The other half deals with other issues related to transgender human rights, like housing and the history of activism. The five videos about prostitution in urban public spaces were produced by free-to-air television networks, four of them of national scope. The other five were produced either by the national public television network, independent media, the Government of the City of Buenos Aires or trans activist organizations. Videos dealing with transgender prostitution have significantly more views (215,000–5,500,000) than those videos dealing with transgender human rights (4–3,524 views). Also, all videos with massive amounts of views were produced by free-to-air television networks. These results show, then, a clear threefold correlation between a specific topic (transgender prostitution in urban public spaces), a particular source (free-to-air television) and a certain (massive) degree of audience.^10^ This correlation can be interpreted as a sign of both the public’s and the mass media’s selective interest in the transgender community.

**TABLE 2:** First ten results of the YouTube search “trans + city + Buenos Aires” (August 6, 2020).

Order	Video title	YouTube channel	Upload date	Views
1	La Ciudad de Buenos Aires deberá garantizar vivienda a tres mujeres trans (The City of Buenos Aires will have to ensure housing for three trans women)	Televisión Pública Noticias (news program, national free-to-air television)	4/30/2020	201
2	* Las chicas trans corridas por los Juegos Olímpicos de Buenos Aires (The trans girls displaced by/because of the Buenos Aires Olympic Games)	Telenoche (news program, national free-to-air television)	9/26/2018	2,088,000
3	Hotel Gondolín: una alternativa de vivienda para travestis y trans en Buenos Aires (Gondolín Hotel: a housing alternative for transvestites and trans in Buenos Aires)	Presentes LGTB (independent international news agency)	12/3/2018	3,524
4	Buenos Aires tiene la primera Casa Trans del país: testimonio de Morena Pinat (Buenos Aires has the first Trans House in the country: Morena Pinat’s testimony)	Prensa GCBA (Government of the city of Buenos Aires’ press channel)	11/16/2017	1,318
5	* VAGABUNDO/Prostitución: La Noche de una trans (VAGABOND/Prostitution: the Night of a trans)	Jujuy al Momento Diario (regional media of the province of Jujuy)	8/20/2019	215,000
6	Recorrido por la Historia de la Militancia Trans en Buenos Aires/Noti Trans—Sociedad y Política (A walk around the History of Trans Activism in Buenos Aires/Noti Trans—Society and Politics)	Noti Trans Argentina (independent trans activist organization)	4/19/2018	304
7	* Travestis: Una pasión de ciertos hombres (Transvestites: A passion of certain men)	70 20 Hoy (journalistic program, national free-to-air television)	9/7/2014	5,500,00
8	* Pasa de noche: travestis—Telefé Noticias (It happens at night: transvestites—Telefé Noticias)	Telefé Noticias (news program, national free-to-air television)	5/23/2018	749,000
9	* Zona Roja—Vértigo (Red Zone—Vertigo)	Telefé (national free-to-air television network)	1/23/2014	967,000
10	Conversatorio Ley Integral Trans en Argentina (Dialogue panel Integral Trans Law in Argentina)	RedLacTrans Oficial (international trans activist organization)	8/6/2020	4

From these results, *Telenoche*’s video was selected for analysis because it had the second largest amount of audience, which suggests that it appealed to—or at least interested—a vast number or viewers. The video with the largest amount of audience (“Transvestites: a passion of certain men,” with five and a half million views) was discarded for analysis because of its less recent date (2014).

The YouTube version of *Telenoche*’s report opens with an aerial nocturnal view of the *Bosques de Palermo*.^11^ We see MC, a cisgender male journalist, walking through the streets of the *Bosques* near the Planetarium, where he briefly explains that, due to the upcoming Summer Youth Olympic Games, the transvestite and transgender sex workers had to be temporarily relocated to that area. This is followed by a brief sequence containing parts of the interviews and takes of the workers standing or walking among their clients’ cars. This sequence lasts a minute and acts as a general introduction. Then follows the main part of the report (78% of the video’s total length), comprised of longer segments of interviews with the transgender sex workers and with a cisgender male *remis* ^12^ driver who works in the area. All sequences are heavily edited, with many cuts that affect the interviewees’ speech. Subtitles are provided only when the interviewees talk. The interview segments are separated by short musicalized clips that show the sex workers and the cars in the streets. During the video, three different headlines appear onscreen in the following order: “Corridas por los Juegos” (“Displaced by/because of the Games”), “Por los JJ. OO. de la Juventud corrieron a las chicas trans de Palermo” (“Due to the Youth Olympic Games they displaced the trans girls of Palermo”), and “Las chicas trans, molestas porque las trasladaron a la zona del Planetario” (“The trans girls, upset because they moved them to the Planetarium zone”).^13^ The video ends with a brief editorial remark by MC and a cisgender female journalist, MLS, live from the television studio.

## Linguistic Analysis

In the following subsections, I present the main results of the linguistic analysis of the corpus.

### Categorization and Category Focalization

To start, I present the discursive categories obtained from the SDMLAT (phase 1 of the MCLA). These are:

- *Speaker-Protagonist (S-P)* and *Value Nexus 1 (VN1)*: these two categories develop the argumentative paradigm sustained by the person who is speaking.
- *Actor* and *Value Nexus 2 (VN2)*: these two categories develop the argumentative paradigm attributed to the other participants in the dialogue situation.

These two sets of categories require some further explanation. As the corpus contains dialogues, many nominal and pronominal items refer to participants who are present in the communicative situation. Depending on which participant is speaking, these nominal and pronominal referents are categorized under the *Speaker-Protagonist* or the *Actor*. For example, when MC is talking to a transgender sex worker, lexical items that refer to MC are categorized under the *Speaker-Protagonist*, while those items that refer to the sex workers are categorized under the *Actor*; and vice-versa, when it is a transgender sex worker speaking to MC, lexical items that refer to the workers are categorized under the *Speaker-Protagonist*, while those that refer to MC are categorized under the *Actor*. It is important not to equate the number of participants who are present in the communicative situation with the number of argumentative paradigms. For example, in this report there are eleven participants (two cisgender journalists, eight transgender women and the cisgender *remis* driver), but only two argumentative paradigms: the television program’s, which includes the discourse of both journalists, and the transgender sex workers’, which encompasses the sex workers and the *remis* driver.

There are two other *Actor* categories, which represent subjects and institutions not present in the communicative situation. These are:

- *Actor* and *Value Nexus 3 (VN3)*: these categories correspond to the Government of the City of Buenos Aires. They were inductively named *Government.*
- *Actor* and *Value Nexus 4 (VN4)*: these categories personify different aspects of prostitution, like the clients and prices. They were inductively named *Work*.^14^
- *Space*: this category appears as a macro-category with three properties^15^: *Planetarium* (the new zone assigned to the sex workers), *Palermo* (the zone where they worked before the relocation), and *Alternatives* (possible sites that were discussed during negotiations with the government).
- *Time.*
- *Pragmatic Operator (PO).*
- *Negation.*

**FIGURE 1:**
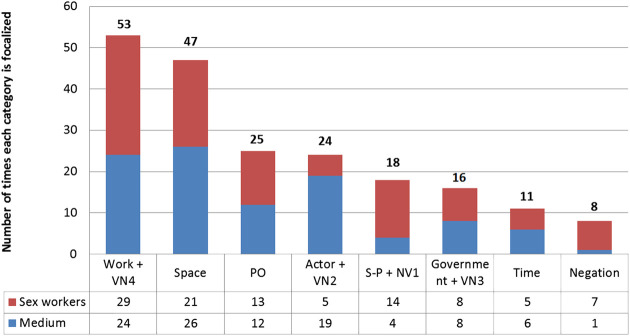
Focus categories.

Figure 1 shows the distribution of the focus categories (phase 2 of the MCLA), illustrating the importance of each category in terms of information hierarchization. The first two bars correspond to the predominant focus categories: the Actor *Work* with its *Value Nexus* and the macro-category *Space*. The graph also discriminates how many times each category is focalized in the television program’s and in the transgender sex workers’ discourse. As we can see, nearly all categories have the same prominence in both cases, with three exceptions: the *Speaker-Protagonist* and *Value Nexus 1* (which are notably more focalized by the sex workers), the *Actor* and *Value Nexus 2* (notably more focalized by the television program), and *Negation* (notably more focalized by the sex workers). These exceptions will be discussed later.

### Prostitution and Urban Space

As I said, the Actor *Work* and *Space* are the two predominant focus categories. In the television program’s discourse, the Actor *Work* is repeatedly focalized to describe, among other aspects of prostitution, the clients. In Table 3,^16^ the questions made by journalist MC focalize the Actor *Work* in relation with the clients’ regularity and their age average. In response, the transgender sex workers (TSW in the examples) also focalize the Actor *Work* and its *Value Nexus*. These categories are reinforced by reoccurring structures (*se repiten/they do repeat*) and lexical choices that add semantic charge (like *las mismas, muchas, nuevas/the same*, *many, new*, used to describe the clients, or the detailed account of age ranges, as in *de veinte años hasta ochenta/from twenty years up to eighty*).

**TABLE 3:** Focalization of the Actor *Work*: the clients.

Id	Time	PO	Actor *Work*	VN4 (Work)
MC		e46[¿**Y**	**los clientes se**	**repiten**
		digamos,	se	ven
	**siempre**		**las mismas caras**?] WF	
TSW4			e47[**Se**	**repiten**
			**muchas caras**.] WF	
MC			e48[**Se**	**repiten**.] WF
TSW4		e49[**Sí**		
	**todas las noches**		un par de **caras nuevas, diferentes clientes, todo nuevo**.] WF	
MC		e50[ Y, pero,	**¿el promedio de edad?**] WF	
TSW5		e51[ Y	**entre veinte y, treinta y cinco**.] WF	
TSW4			e52[**De veinte años hasta ochenta**.] WF	
MC		u46[**And**	**the clients,**	**do**
			**they**	**repeat**,
		I mean,		
	(you) **always** (see)		**the same faces**?] WF	
TSW4			u47[**They**	**do repeat,**
			**many faces**.] WF	
MC			u48[**They**	**do repeat**.] WF
TSW4		u49[**Yes**		
	**every night**		a couple **new faces, different clients, everything new**.] WF	
MC		u50[And, but,	**their age average?**] WF	
TSW5		u51[And	**between twenty and, thirty-five**.] WF	
TSW4			u52[**From twenty years up to eighty**.] WF	

The television program also directs its attention to the economic aspects of prostitution. Most of MC’s questions and comments emphasize the effects of the economic crisis and the inflation on the workers’ income. This includes comparing their current situation in the Planetarium area with their previous location, especially regarding the amount of clients. Table 4 shows three interventions made by MC in different moments of the report. Utterance 20 belongs to the brief explanation he gives during the video’s introduction, while utterances 120 and 160 are questions that he asks, respectively, to the *remis* driver and a transgender sex worker.

**TABLE 4:** Focalization of the Actor *Work*: economic aspects. 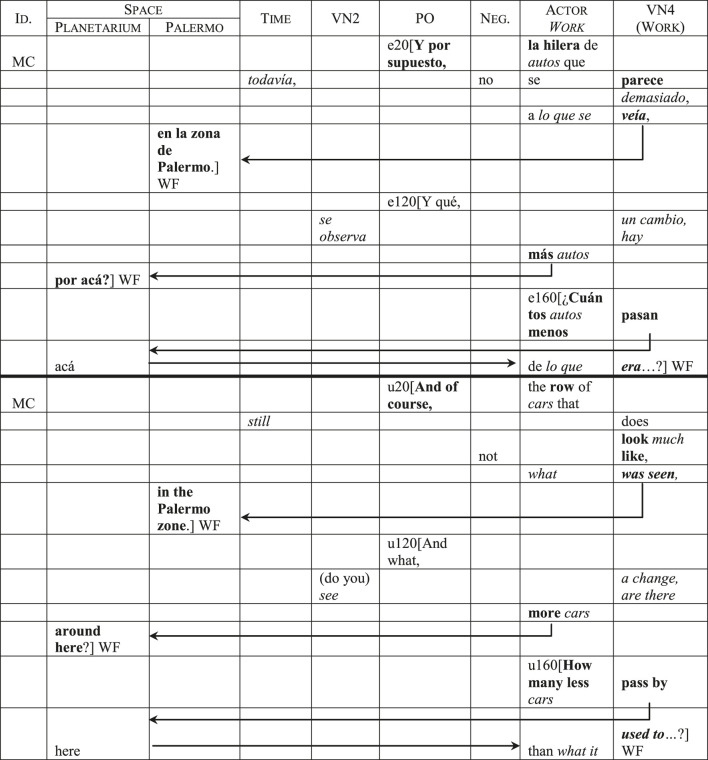

The first thing I wish to point out in this example is that the Actor *Work* and its *Value Nexus* are, at the same time, reinforced and mitigated. On the one hand, the quantitative elements of the comparison are reinforced by adverbs like *más* (*more*), *menos* (*less*) and *demasiado* (*much*), by lexical choices like *hilera* (*row*), which suggests a certain quantity and disposition, and by the contrastive use of the present and past tenses in *se parece* and *veía* (*look like/was seen*). On the other hand, other concrete aspects of the change that supports the comparison are mitigated. This mitigation affects both the Actor *Work* and its *Value Nexus*: in the first case, the lexical choice *autos* (*cars*), which constitutes a metonymy (the vehicle for the driver), and the use of the neuter pronoun *lo* (*what*) blur the reference to the concrete individual clients; in the second case, the use of the passive voice (*se veía/was seen*), nominalizations (*un cambio/a change*), and existential processes (*hay*, *era/are there*, *used to*) dilute the semantic charge of the actions and states associated with this *Actor*.

The second thing I want to point out are the categorical displacements^17^ that lead from the Actor *Work* and its *Value Nexus* to the *Space* category. In utterances 20 and 120, these displacements lead to a halt in the production of discourse, making *Space* the focus category of the utterance (see the “WF” flags). In utterance 160, a subsequent displacement goes “back” from *Space* to the Actor *Work*, which provides the second element of the comparison (*de lo que era/from what it used to*) and becomes the focus category. This illustrates how the Actor *Work* and *Space* become the report’s two predominant focus categories, placing cognitive emphasis on the changes in geographical location and their impact on the sex workers’ activity.

In this regard, the diachronic observation of the macro-category *Space*—which shows how it is semantically charged throughout the text by the reiteration of items that constitute lexical fields (Pardo, 2011, p. 86)—shows interesting differences depending on the participant. While the journalists’ discourse about urban public space contains more “objective,” non-evaluative terms, the transgender sex workers use more evaluative terms that explicitly involve subjectivity. This contrast is exemplified in Table 5. As can be seen, the semantic construction of the *Space* category in the television program’s discourse includes proper names for places, buildings and streets, as well as common nouns like *zona* (*zone*), *barrio* (*neighborhood*) and *destino* (*destiny*), to describe the neighborhood and the park as the setting of the report, and to refer to the alternatives that were discussed during negotiations with the city’s government. On the other hand, the transgender women use adjectives like *chiquitito (super tiny*), *nefasto* (*terrible*) and *horrible* to reinforce negative characteristics of the Planetarium area and its alternatives in terms of their disadvantages for prostitution; but also adjectives like *potable* (*acceptable*) and *positivo* (*positive*), to express their hopes of finding a solution, and constructions like *un orgullo nacional* (*a national pride*), to appraise positive aspects of the Planetarium.

**TABLE 5:** The semantic construction of the *Space* category in the discourse of the television program and the transgender sex workers.

Non-evaluative terms in the television programs’s discourse	Evaluative terms in the transgender sex workers’ discourse
A la derecha el Planetario/esta zona, este barrio, este territorio/en la zona de Palermo/el lugar/otras alternativas/la opción Planetario/el Rosedal/el primer destino/ahí al bosque/el predio/la calle Belisario Roldán/casi dos cuadras. To the right the Planetarium/this zone, this neighborhood, this territory/in the Palermo zone/the place/other alternatives/the Planetarium option/the Rose Garden/the first destiny/there to the woods/the land/Belisario Roldán Street/almost two blocks	Lo que es esto, re chiquitito/un orgullo nacional el Planetario/nos cultiva de un modo increíble/un asco/era nefasto/alguna solución potable/algo positivo/está horrible/mucha villa/es muy chiquita la zona. What this is, super tiny/the Planetarium, a national pride/it cultivates us in an incredible way/gross/it was terrible/some acceptable solution/something positive/it’s horrible/much slum/the zone is very tiny

### The Socio-Discursive Representation of the Transgender Sex Workers in Relation With Urban Space and Prostitution

The two predominant focus categories, the Actor *Work* and *Space*, have an important role in shaping the socio-discursive representation of the transgender women. In the television program’s discourse, this is shown by the categorical displacements that systematically connect those two categories with the *Actor*. Concerning *Space*, this involves verbs that express either *moving* through space, *existing* in space or even *being a part of* space. Table 6 shows three utterances from MC. Utterance 19 belongs to his opening explanation, while utterances 28 and 158 belong to his conversations with two transgender women. The arrows indicate the displacements from the *Value Nexus 2* towards *Space*, which in all cases is reinforced as the focus category. In utterance 19, the action in *Value Nexus 2* (*empiezan a formar parte/start being a part of*) associates the transgender women with space in terms of identification: their relocation implies that they stopped *being a part of* a particular area of the park and began *being a part of* another. Here the *Space* category is reinforced not only by its focus position, but also by the semantic charge added by lexical choices *zona* (*zone*)*, barrio* (*neighborhood*) and *territorio* (*territory*). The repetition of the adjective *este/esta* (*this*), which connotes proximity, also reinforces the *Space* category. In utterance 28, the action in *Value Nexus 2* is expressed by the verb *to be* (*son/are*), associating the transgender women with *Space* in terms of their quantified existence: MC asks *how many* girls are currently in that space.^18^ Finally, in utterance 158, the action in *Value Nexus 2* is expressed by the more dynamic verb *van a volver* (*you will return*), which associates transgender women to *Space* in terms of physical movement, thus presenting them with a higher degree of agency.

**TABLE 6:** The representation of the transgender sex workers in relation with *Space*. 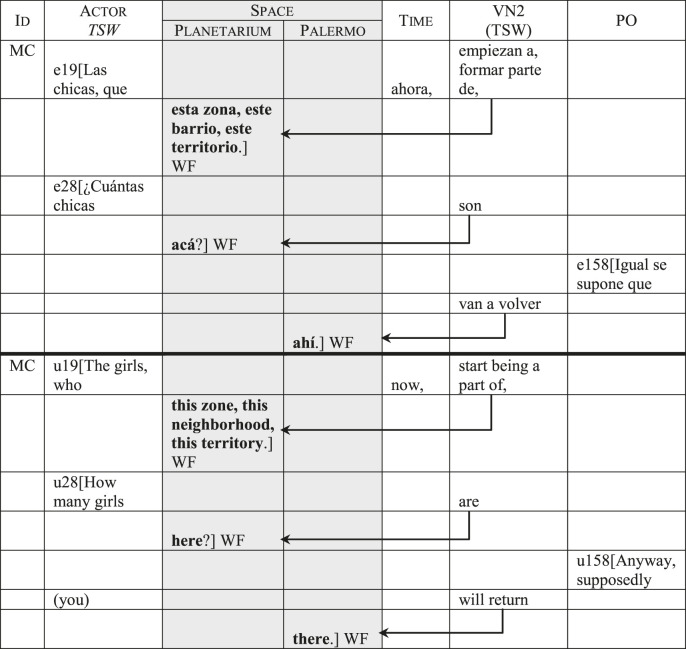

In some cases, lexical items referring to space are used metonymically, to allude to the activity (prostitution/sex work) that occurs in that space. This results in a discursive identification of the Actor *Work* and *Space* categories.^19^ In Table 7, which corresponds to the closing editorial remark by MC, the items *acá* (*here*) and *en otro lado* (*somewhere else*) have a primarily spatial reference. However, the context shows that these adverbial elements are being used to talk about something more than geographical location: when MC says that the transgender sex workers would rather be *somewhere else*, he is implying that they would prefer having a different type of occupation. In the example, these lexical items were duplicated in the Actor *Work* and *Space* categories^20^ to illustrate the resulting discursive identification. An important effect of this is that the transgender women’s identities are symbolically defined in the narrow terms of those particular urban spaces and the activities that take place in them.

**TABLE 7:** Discursive identification of the *Space* and Actor *Work* categories. 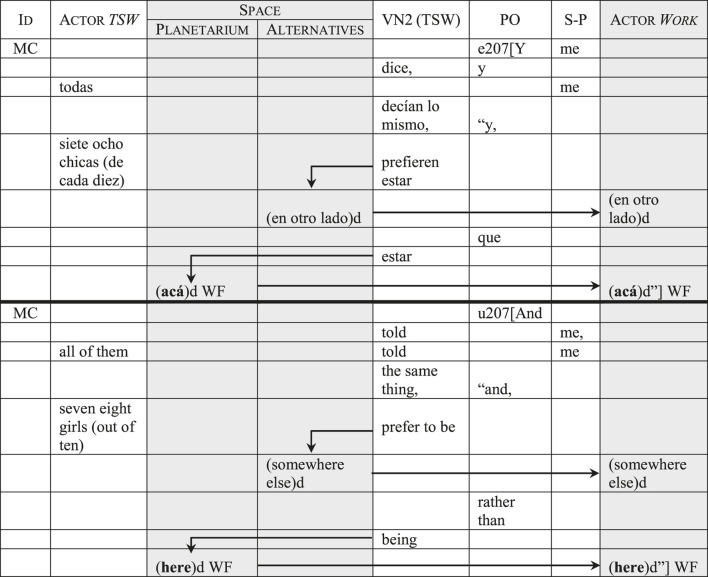

The transgender women’s discourse presents a different approach to the *Space* category than the television program. Table 8 shows the process types that express the sex workers’ actions and states related to urban space. In this regard, there is a significant contrast in the predominant process types: while the television program favors relational processes (40%)—which, as we saw, define the sex workers’ interaction with space in terms of mere *being*–, the vast majority of the processes used by the transgender women (63%) are material, describing a more active interaction with urban space in terms of *working*, *looking for* alternative areas, etc. Although the program also uses a significant amount (33.3%) of material processes to talk about the workers’ relation with space, these actions are circumscribed to physical mobility, with the workers being presented as *coming*, *going* or *returning* to different areas of the park.

**TABLE 8:** The transgender sex workers’ actions and states (in bold) related to the *Space* category (in italics).

In the television program’s discourse		
Process type	Examples	Frequency
Relational	(…) por un mes **van a estar** *aquí* (for a month they **will be** *here*)	40%
Material	Igual se supone que **van a volver** *ahí* (Anyway, supposedly you **will return** *there*)	33.3%
Mental	¿Les **gustaba** *el Rosedal*? (**Did** you **like** *the Rose Garden*?)	20%
Existential	¿Cuántas chicas **son** *acá*? (How many girls **are there** *here*?)	6.6%
**In the transgender sex workers’ discourse**		
**Process type**	**Examples**	**Frequency**
Material	Nosotras *acá* **trabajamos** con nuestro cuerpo (We **work** *here* with our body) (…) **llegamos a**, bueno **a, a buscar** *alguna solución potable* (we **came to**, well, **to, to look for** *some acceptable solution*)	63.6%
Relational	(…) *donde* **estábamos** antes nos pusieron cámaras (*where* we **were** before, they set cameras)	36.3%

### The Socio-Discursive Representation of the Transgender Sex Workers in Relation with the Government of the City of Buenos Aires

The Government of the City of Buenos Aires is represented by an *Actor* with its corresponding *Value Nexus (VN3)*. In the discourses of both the television program and the transgender sex workers, several linguistic resources converge in those categories to produce a socio-discursive representation of the government that is closely related to the representation of the transgender women.

In the television program’s discourse, the categorical displacements systematically connect the Actor *Government* and its *Value Nexus* with the categories of *Space* and the *Actor* that represents the sex workers. These displacements reinforce the government’s role as an agent that efficaciously controls public space and its occupants. Table 9 illustrates this with an interview segment. In utterance 21 (a comment made by MC to trigger dialogue), the first displacement goes from the *Actor* that represents the sex workers, instantiated by the object pronoun *las*, to the government’s *Value Nexus*, instantiated by the action *cambiaron* (*changed*), and from there to the focus category *Space*, instantiated by the prepositional construction *de lugar* (*your place*).^21^ The same pattern appears in the first part of the sex worker’s response (utterance 22): starting from the object pronoun *nos* in the *Speaker-Protagonist* category, discourse moves towards the same action (*cambiaron/changed*) in the government’s *Value Nexus*, and from there to the same prepositional construction (*de lugar/our place*) in the *Space* category. Then, after passing through the *Value Nexus 1* and *Time* categories, the same pattern—an object pronoun in the *Speaker-Protagonist* followed by an action in the government’s *Value Nexus*—is repeated twice to indicate governmental vigilance (through cameras and reflectors) aimed at thwarting the return of the transgender sex workers to their usual area in the park. Finally, in utterance 23, the same displacement pattern appears yet two more times (*nos dijeron, nos quieren dejar/they told us, they want to leave us*) and the utterance ends with *Space* as the focus category. The constant repetition of this displacement pattern reinforces the government’s agency over the transvestites and transgender women, who in turn are represented as passive recipients of those actions through the reinforcing reiteration of the object pronouns.^22^

**TABLE 9:** The representation of the Government of the City of Buenos Aires. 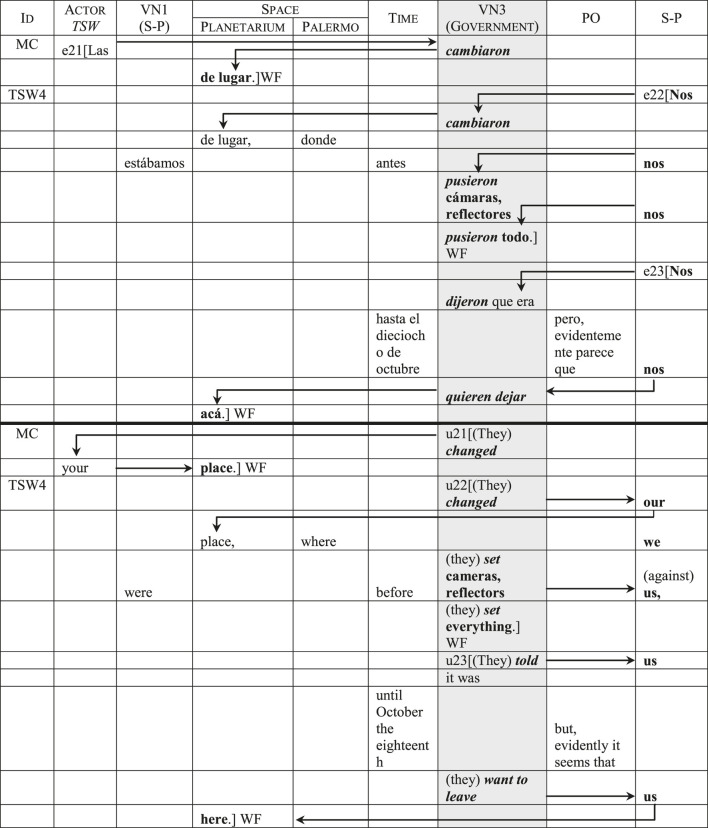

The reinforcement of the Government of the City of Buenos Aires as the controller of urban space coexists with several mitigating resources that omit or conceal it as a *concrete* agent. These resources can be observed both in the television program’s and in the transgender women’s discourse, but they are more frequent in the former, especially in MC’s questions and comments. Table 10 classifies and exemplifies these mitigating resources. First, we have the erasure of the grammatical subject (see utterances 35 and 209 in the table; for additional examples, see all instances of the *Value Nexus 3* in Table 9). Unlike English, in Spanish the subject can usually be omitted because the verbal desinence indicates tense, number and person. Erasing the grammatical subject can thus be used to avoid specifying the identity or nature (in this case, the governmental nature) of the agent. In the second place, we have nominalizations: in utterances 136 and 216, the nominal forms *el cambio* (*the change*) and *la reubicación* (*the relocation*) avoid explicitly stating *who made* the change or *who relocated* the transgender women. The same can be said of passive forms: in utterances 78 and 140, the passive forms with the pronoun *se* avoid specifying *who negotiated* or *who said* certain things during negotiation. In utterance 143, the past participle *pensado* (*conceived*) could be followed by a complement that specifies the agent (conceived *by …* ); its absence constitutes another mitigating resource. The following mitigating resource is using impersonal forms: in utterance 3, the Spanish forms *haber* and *haber que* allow to present the acts of relocating the transgender women and negotiating with them as impersonal events that simply “take place,” as if without any concrete agent’s intervention.

**TABLE 10:** Mitigating resources that affect the Government of the City of Buenos Aires as an agent.

Resource	Examples (resources in italics)
Erasure of the grammatical subject	¿Les *dieron* un subsidio, les *dieron* algo? *Did* (they) *give* you a subsidy, *did* (they) *give* you anything? (u35) Desde la ciudad me *dijeron* que, cuando terminen los juegos van a volver adonde estaban. From the city (they) *told* me that, when the games are over, they will go back to where they were. (u209)
Nominalizations	¿Cómo cayó *el cambio* de lugar? How was *the change* of place received? (u136) Una de las consecuencias que, colaterales que trajeron los juegos olímpicos, la *reubicación* de las chicas. One of the collateral consequences that the olympic games brought [was] the *relocation* of the girls. (u216)
Passive forms	¿*Se negoció* rápido el lugar? *Was* the placed *negotiated* fast? (u78) ¿En esas tres reuniones en la primera qué *se dijo*? In those three meetings, what *was said* in the first one? (u140) Al Rosedal, ese era el primer destino *pensado*. To the Rose Garden, that was the first *conceived* destination. (u143)
Impersonal forms	Donde antes paraban, las chicas trans, en lo que se llama habitualmente zona roja, *hubo que trasladarlas*, *hubo una negociación* y, por un mes van a estar aquí. Where the trans girls used to be, in what was usually called the red zone, *they had to be moved*, *there was a negotiation* and, for a month they will be here. (u3)
Presenting the reason for the displacement as the agent	Corridas *por los Juegos*. Displaced *by/because of the Games*. (Headline 1) Una de las consecuencias que, colaterales que *trajeron los juegos olímpicos*, la reubicación de las chicas. One of the collateral consequences that *the olympic games brought* [was] the relocation of the girls. (u216)
Use of the *Space* category	¿Y *el Planetario cómo aparece como opción*? And *how does the Planetarium appear as an option*? (u155)

So far, these resources affect the *Value Nexus 3*, that is, the category that expresses the government’s actions and states. The last two rows in the table show other categories that are used to mitigate the government’s agency. In the first headline that appears onscreen (on which the title of the YouTube’s video is based), the Spanish preposition *por* in *por los Juegos* can be read both as indicating the agent (*displaced by the Games*) or the reason (*displaced because of the Games*); while in utterance 216, *los juegos olímpicos* (*the olympic games*) are explicitly constructed as the grammatical subject and agent. These constitute two cases of presenting the reason adduced for the relocation as the agent itself, which of course contributes to hiding the actual agent: the city’s government. Finally, as we can see in the last row (utterance 155), the *Space* category is used to describe the Planetarium as an option that, during negotiations between the transgender collective and the government, “appeared” as if by itself.

In the transgender sex workers’ discourse, the Actor *Government* generally exhibits the same reinforcing and mitigating resources. This is especially noticeable in the object pronouns and categorical displacements that reinforce the transgender women as passive recipients of the government’s actions, and in the constant erasure of the grammatical subject that mitigates the government’s agency (see all instances of the *Speaker-Protagonist* and *Value Nexus 3* categories in Table 9). However, the transgender women also use (a few) reinforcing resources to highlight the government’s violence against them. In Table 11, utterance 26, the transgender woman explains that they cannot return to their usual working areas because, if they do, the police immediately detain them. Although the action *llevan presa* (*throw in jail*) is mitigated by the erasure of the grammatical subject, it nevertheless closes the utterance, making the government’s *Value Nexus* the focus category and thus reinforcing institutional violence. The *Pragmatic Operator* category is also used to reinforce this violence by adding a sense of instantaneity: going to their usual zone amounts to being detained *straight away*. In utterances 202 and 203, another transgender woman describes the treatment they receive from the government in terms of not being regarded as *people*. She focalizes the Actor *Government* and the *Speaker-Protagonist* categories to respectively reinforce the one who denies them their subjectivity (*para ellos*/*for them*), and the denied human condition (*personas*/*people*). This is the only focalization in the whole video that personifies the Actor *Government*, even if it does so vaguely, with a third person pronoun.

**TABLE 11:** Reinforcement of institutional violence against transgender women.

Id	Actor *Government*	VN1 (S-P)	Space	VN3 (Government)	PO	Neg	S-P
			**Palermo**				
TSW4					e26[Y		
			los lugares que				
		podías estar,			**ya directamente**		te
				***llevan* presa**.] WF			
TSW8							e202[Te
				*tratan* como, como, como			lo que
						**no**	
		somos,			*supuestamente*,		
	**para ellos**.] WF						e203 [**Personas**.] WF
TSW4					u26[And		
			the places where				(you)
		could be,					
	(they)				**straight away**		
				**throw**			you
				**in jail**.] WF			
TSW8	u202[(They)			*treat*			you
				like, like, like			what we
		are				**not**,	
					*supposedly*,		
	**for them**.] WF						u203 [**People**.] WF

Finally, the transgender women’s discourse also presents a distinctive use of the *Negation* category to reinforce the government’s inaction and neglect of their collective. Table 12 shows how their answers to MC’s question reinforce the *Negation* category by repeating the lexical item *nada* (*nothing*, *anything*) which affects the actions expressed in the government’s *Value Nexus*. It is worth noticing that here, just like in Table 9, all categorical displacements present the transgender women as passive recipients of the government’s actions (or lack thereof).

**TABLE 12:** The *Negation* category in the transgender sex workers’ discourse. 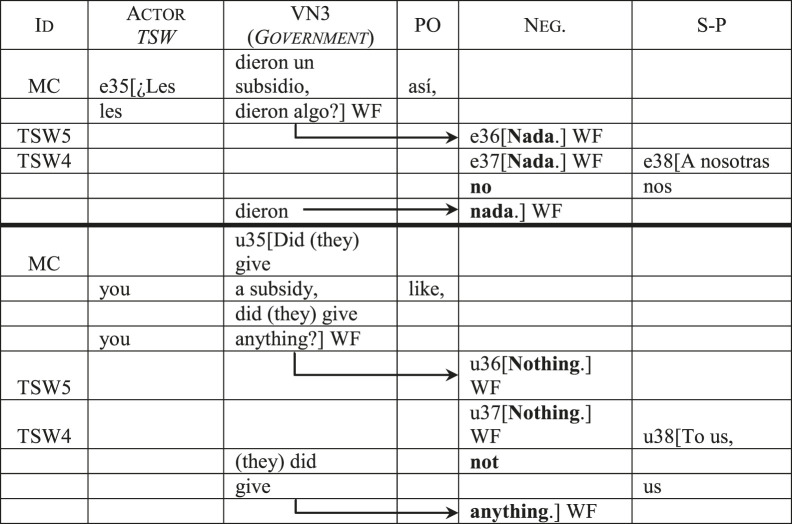

### The Control of Discourse

According to van Dijk (1993, 1999, 2001a), social power is directly related with the degree of control that different participants have over the properties of discourse and its context: members of more powerful social groups tend to have more control over textual and contextual aspects of discourse, and vice versa. In the case here under analysis, historical and political factors—such as the history of the Clarín media group and deeply rooted discrimination of non-traditional gender identities in Argentina—make one expect a higher degree of social power from the television program in relation to the transgender sex workers. In that regard, my analysis of this report provides linguistic evidence of this power asymmetry and of its effect on the construction of discourse.

In the first place, I have noted that, although the distribution of focus categories is similar in the discourses of the television program and the transgender women (see Figure 1), there were some exceptions. Two of them involved the *Actor* and *Value Nexus 2* categories—which are notably more focalized by the program –, and the *Speaker-Protagonist* and *Value Nexus 1* categories—notably more focalized by the transgender women. As I have explained, in the program’s discourse the *Actor* and *Value Nexus 2* categories represent the transgender women; while, in the transgender women’s discourse, the *Speaker-Protagonist* and *Value Nexus 1* represent the women themselves. This asymmetry shows that, regardless of who is speaking, the transgender women are always the most focalized participants, coming into the spotlight only to be “talked about” in terms of their quantity, spatial location, and the economic details of prostitution. In contrast, the television program’s discursive position remains consistently out of the spotlight.^23^ The analysis of information focalization, then, shows the program’s greater degree of control over the exposure of the different participants.

The same analysis also shows that the television program controls which discursive categories are emphasized through the editing process. This happens in two ways. The first involves the editing cuts that, during the whole video, truncate what a person is saying, with the presumable purpose of leaving out pauses or interruptions and abbreviating their speech. Whichever the intended purpose, these cuts have a concrete impact on the distribution of focus categories as they create new focuses. There are at least ten of these cuts in the video, and all of them affect the transgender sex workers’ discourse. The focuses created by these cuts correspond to the following categories: the Actor *Work* (30%), *Space* (30%), the Actor *Government* (20%), the *Speaker-Protagonist* (10%), and the *Pragmatic Operator* (10%). This means that, in the case of the two predominant focus categories (Actor *Work* and *Space*), the editing cuts account respectively for 5.4 and 6% of the total focuses.

The second way in which the editing process impacts on information focalization involves the short compilation of interview segments that opens the video. These segments are repeated later, resulting in nine utterances that appear twice. Consequently, nine particular focuses are repeated, placing an extra emphasis on certain discursive categories. These are: the *Value Nexus* that corresponds to the transgender sex workers (44.4%), the Actor *Work* with its *Value Nexus* (33.3%), *Space* (11.1%) and *Time* (11.1%). Regarding the first two categories, focus repetition produced by editing accounts respectively for 22.2% and 5.4% of the total focuses. The information thus highlighted is always related to the practical and economic aspects of prostitution. Therefore, by creating and repeating focuses in the editing process, the program shapes the general distribution of focus categories and reinforces the topics of urban space and prostitution as were described in *Prostitution and Urban Space* and *The Socio-Discursive Representation of the Transgender Sex Workers in Relation With Urban Space and Prostitution*.

Finally, in the closing editorial remark by MC and MLS, as is traditional, the program has the final word on the subject. Here, both cisgender journalists bring up the fact that transgender women experience greater difficulty in getting jobs than cisgender people, and that most of them would prefer to leave prostitution. In Table 13, utterance 207, MC expresses this view by reporting the transgender women’s words. This utterance was already displayed in Table 7, to show how urban space and prostitution become discursively identified through the categories *Space* and the Actor *Work*. In the following utterance (208), MC reinforces his assertion about transgender people’s exclusion from the labor market by describing their working conditions in the public space. In doing so, he deviates from the program’s predominant use of non-evaluative terms in the *Space* category (see Table 5) to introduce evaluative terms that reinforce the precarious spatial (*a la intemperie/out in the open sky*) and temporal (*en el medio de la noche/in the middle of the night*) conditions. However, despite these sympathetic remarks, the following and conclusive utterance (209) returns to the discursive identification of urban space and prostitution as the symbolic place where the transgender women will *go back* once the Olympic Games have finished. This way, the complex issue of structural transgender exclusion—which was barely addressed during the interviews—is screened by the “news” that the transgender women will eventually be permitted to return to their previous location. As a result, the *status quo* is naturalized and presented as the end of a conflictive situation.

**TABLE 13:** The naturalization of social exclusion in the closing editorial remark. 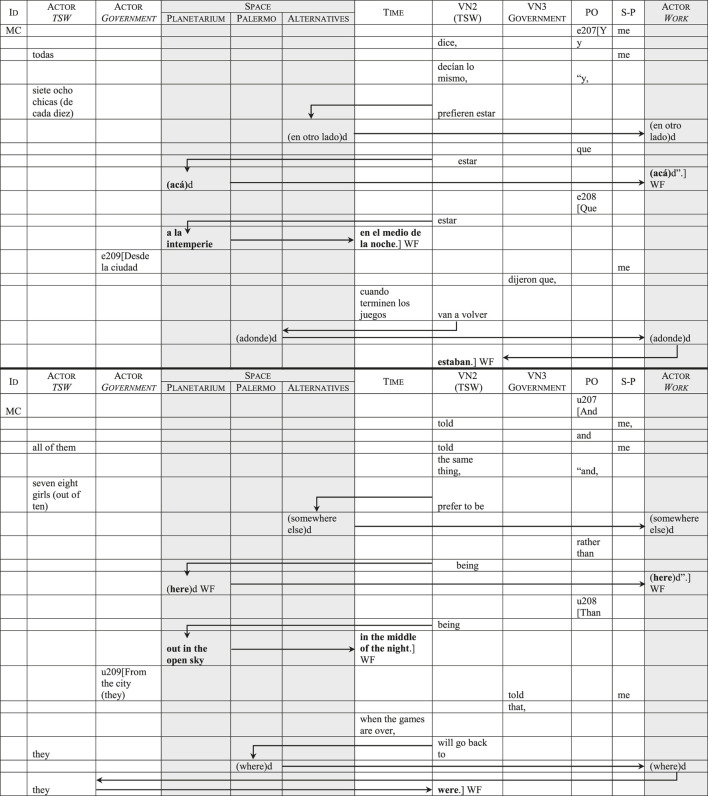

## Conclusion

The linguistic analysis revealed some significant traits of the socio-discursive representation constructed in this news report:

- The cognitive emphasis is placed on prostitution and urban public space, with the Actor *Work* and *Space* as the predominant focus categories. These categories highlight different aspects of prostitution, especially the economic (clients and prices), in relation to the changes brought by the geographical relocation. Quantitative changes (client frequency, inflation) are reinforced, while the clients’ concrete identities and agency are mitigated.
- The television program’s representation of urban public space is constructed mainly with non-evaluative terms that refer to geographical location. The transgender sex workers are portrayed merely as circulating bodies or, sometimes, even as another part of public space. In contrast, the transgender women describe their relation with public space not only in terms of circulating and being, but also of working. In this sense, they appraise different places with evaluative terms regarding their safety, appearance, etc.
- The television program uses adverbial elements connected with space and names like “red zone” in an ambiguous metonymical sense that produces a discursive identification of urban public space and prostitution. These ambiguous elements are in close connection with the categories that represent the transgender women, resulting in the symbolical reduction of their identities to the “prostitution/public space” pair.
- The Government of the City of Buenos Aires is represented by the television program as an agent that efficaciously controls the transgender women’s occupation of public space. At the same time, the specificity of this governmental agent is systematically mitigated by different linguistic resources that allow concealing or omitting the grammatical subject. This linguistic strategy allows highlighting the effects of governmental control while avoiding any concrete reference to their context, such as the concrete participants, the history and the dynamic of the negotiation behind the relocation, etc. Governmental actions are thus discursively represented as impersonal events.
- Only the transvestites and transgender women focalize the Actor *Government* and *Negation* categories to address institutional violence and the neglect of their work and life conditions.
- Both through the dialogues and the subsequent editing process, the television program exerts a high degree of control over the properties of discourse and the communicative situation. This is signaled by the tendencies found in the analysis of information focalization in relation with the discursive traits described in the previous points.

From these points, I conclude that this news report is pervaded by a discursive naturalization of the *status quo*. While the transvestites and transgender women are presented as passive, consumable bodies that circulate in and make up urban public space, the Government of the City’s restrictive actions to order that space—and the bodies that inhabit it—appear as effective yet impersonal events. Although the journalists mention that most transgender women would prefer to have a different occupation, the report’s general focus consolidates the idea of prostitution as their “natural place,” with the closing editorial remark describing normality as “going back to where they were”. To use a theatrical metaphor, the report’s discursive stage is crowded with different *Actors* that represent the transgender women, the Government, the clients and even the economic crisis. However, there is no scenery: no semantic-discursive categories are used to provide background or context. The spotlight falls on the geographical relocation, but the negotiation process that led to it, the role of the transgender movement and the structural exclusion are left out of the spotlight and behind the curtains. For this reason, even though this television piece is politically correct and does not display any evident form of gender-based violence, the socio-discursive representation it constructs sustains, through its de-contextualizing and naturalizing effects, a degree of symbolic violence (Bourdieu, 1998). This symbolic violence is discursively epitomized by the closing description of the transgender women’s displacement as a mere “collateral consequence” of the Olympic Games (see utterance 216 in Table 10), which does not only appraise the women’s situation as less important than the realization of the Games, but also tacitly bases this appraisal on the fact that, sooner or later, they will return to their “natural place”.

These results do not differ essentially from those of previous research into the socio-discursive representation of transvestites and transgender women in similarly themed television pieces from the decades of 1990–2000 (Soich, 2010; Soich, 2011; Soich, 2016). By 2018, the social visibility of transgender issues had increased notably, and much reliable information about transgender people’s lack of access to basic human rights in Argentina had been produced. However, the report’s lackluster conclusion about the transgender women going “back to where they were” makes one think that it is rather the mainstream media who have yet to depart from their old habits.

## Data Availability

The original contributions presented in the study are included in the article/Supplementary Material, further inquiries can be directed to the corresponding author.

## Footnotes

1 In the feminist and gender diversity movements in Argentina, there is an intense debate about whether prostitution is a form of sexual slavery and exploitation, or a job that must be legally acknowledged and protected by the State. Those who support the first position use the term “people in prostitution situation,” while those who support the second use the terms “sex work” and “sex worker/s” (Berkins and Korol, 2007). For methodological reasons (see footnote 14), here I use “sex worker/s” to refer to the transvestites and transgender women who appear in the news report. To refer to the activity, I use “prostitution” in a broad sense.

2 75.7% of the interviewed transvestites and transgender women had turned to prostitution at 18 or less; 30% had begun at ages 11–13 (Ministerio Público de la Defensa and Bachillerato Popular Travesti-Trans Mocha Celis, 2017, pp. 58–61).

3 Located in the north of the city, Palermo is its biggest neighborhood and green area, with many transportation connections, shops and tourist sites. Most of its inhabitants have middle and high-level incomes.

4 The situation described in this section coexists with significant legal and social advances in recent years, such as the passing of the law that enabled same-sex marriage in 2010, the passing of the National Gender Identity Law in 2012, several local laws and a recent presidential decree that established a transgender work quota in the public administration, the inclusion of transgender identities in the law that legalized abortion (which contains the terms “pregnant persons” and “women and persons with other gender identities with the capacity to conceive”), and an increasing visibilization of transgender issues in the media and social debate; including the designation, in 2019, of transgender activist Alba Rueda as the first Subsecretary of Diversity Policies in the new National Ministry of Women, Gender and Diversity. The coexistence of these transcendental advances with the inexcusable life conditions and hate crimes suffered by the transgender community defines what Rueda has called “the non-linearity of the transgender political subject” (Rueda, A., “Personas Trans: lecturas sobre pobreza, diversidad sexual y estrategias políticas en Sur América,” lecture, X Congress of the Latin American Network for the Analysis of the Discourse of Extreme Poverty, Buenos Aires, November 10, 2015).

5 According to Pardo (1996, 2011), an utterance is a unit for the analysis of language in use that is lesser than discourse. An utterance is defined by the concurrence of three criteria: pauses and intonation in oral speech, the presence of punctuation marks in writing and the full realization of a theme and a rheme, in both.

6 The MCLA has three more phases, which respectively study the roles attributed to different participants, the use of metaphorical expressions and aspects connected with the discursive genre and context (the last one is currently in development). For more information, see Marchese (2011, 2012, 2016), Soich (2017) and Pardo et al. (2018, in press).

7 In this case, without further context, the Spanish preposition *por* can mean both “because of” and “by”.

8 Today, this group owns multiple companies that produce and distribute contents all across Argentina: newsprint, newspapers, magazines, radio stations, television channels and film studios, and internet and telephone companies. It thus dominates a very significant part of the country’s media and communication market. For more information, see Sivak (2013), Becerra (2019) and Zaiat (2020).

9 Report available at: https://youtu.be/IECOD6uGanU (last accessed January 17, 2021). When last accessed, the video had new comments made only a day ago.

10 The case of the first video in the table suggests that the topic is central in this correlation: though produced by the public national free-to-air television network—which could lead to expect a significant audience–, this video had only 201 views. Significantly, it is the only video produced by a television network that does not deal with transgender prostitution.

11 In this paragraph I offer a minimal description of the audiovisual components of the report. My analysis focuses on verbal text, with the exception of three written headlines mentioned below, which were analyzed through the MSDALT and MCLA. The relation of image and text was only considered indirectly, in relation with information hierarchization (see *The Control of Discourse*). A full multimodal analysis that takes into account image, music and sound was not conducted for this work.

12 A *remis* is similar to a taxi, but it can only be called by phone and charges a flat rate according to the travelled distance.

13 Like the report’s title, the first two headlines mention the Olympic Games as the cause of the transgender women’s relocation, which implies that the realization of the Games is a valid reason for displacing the women from their usual location in the park. In *The Socio-Discursive Representation of the Transgender Sex Workers in Relation With the Government of the City of Buenos Aires*, we will see that this mention of the Games also constitutes a linguistic resource aimed at mitigating the Government’s agency in that displacement.

14 The SDMLAT is a fundamentally inductive method. This means that the socio-discursive representations are defined by the discursive categories that emerge from the text. Therefore, the classification and naming of these categories should respect the text’s materiality as much as possible (Pardo, 2011, pp. 66–67). Throughout this news report, the transvestites and transgender women talk about their activity using terms like “work,” “job” and “sector,” but not “prostitution”: for this reason, the corresponding emerging category was named *Work*.

15 A macro-category is a category (grammaticalized or semantic-discursive) that encompasses other categories, which are considered its properties (Pardo, 2011).

16 In all SDMLAT examples, the cells must be read from left to right and from top to bottom. Each table is divided in two: the upper half shows the analysis of the Spanish original and the lower offers an approximate English translation. The column “Id” indicates which participant is speaking. Utterances appear between brackets and are preceded by the letter *u* (in Spanish, *e* for *emisión*) and their number. The letter combination “WF” indicates focus categories (Marchese, 2011, p. 38). Reinforcing resources are marked in bold and mitigating resources are marked in italics. In some examples, the most important columns were highlighted in grey.

17 The theory of categorical displacements is “a systematized study of the movements made by the speaker within the utterances when passing from one category to another” (Pardo, 2011, p. 99). This theory allows us to observe how a speaker “moves” through different semantic contents and, therefore, what displacement patterns are established between these contents in the unfolding of discourse. In all examples, categorical displacements are illustrated by arrows.

18 The portrayal of transvestite and transgender sex workers as quantities that occupy urban space has appeared in previous analyses (Soich, 2016).

19 The use of the *Work* category to allude to different aspects of prostitution and its discursive identification with the *Space* category trigger the question as to whether *Work* could be considered a semantic-discursive category rather than an *Actor*. This is indeed an interpretative possibility allowed by the theory, as, on the one hand, discursive representations can be instantiated in grammaticalized as well as in semantic-discursive categories (Pardo, 2011, p. 82); and, on the other hand, grammaticalized categories can assume semantic features that strengthen their argumentative functions (Pardo, 2015). In this report, the *Work* category assumes specific semantic features and is clearly a central part of the discursive representation of prostitution. However, I have chosen to interpret it primarily as a grammaticalized category –an *Actor*–, because its main argumentative function is to represent the internalized social voices of the clients and their interest, as part of a greater “voice” that embodies the prevailing economic conditions and their influence on the transgender workers’ activity.

20 Duplicated lexical items always appear between brackets, followed by the letter *d*.

21 In this example, it was impossible to offer an accurate English translation while, at the same time, respecting the Spanish pattern of categorical displacements. For this reason, although that pattern was maintained as much as possible, the arrows in the two halves of the table do not match.

22 Utterance 22 is an exception, because the object pronouns *nos* (*nos pusieron cámaras*, *nos pusieron todo*) are not functioning there as direct objects but as “datives of interest”: they indicate that the actions of *setting cameras* and *setting everything* are (negatively) affecting the sex workers’ interest.

23 The only exception is a brief “playful” discussion between MC and two transgender sex workers, about whether MC fits with the average age and physical appearance of the regular client.
